# Supplementary material for: ARS2/MAGL signaling in glioblastoma stem cells promotes self-renewal and M2-like polarization of tumor-associated macrophages
Source: Nat Commun. 2020 Jun 12;11:2978. doi: 10.1038/s41467-020-16789-2 (PMC7293269; doi:10.1038/s41467-020-16789-2)
Supplement: Supplementary file 1 — Supplementary information [file 41467_2020_16789_MOESM1_ESM.pdf]

SUPPLEMENTARY INFORMATON

ARS2/MAGL signaling in glioblastoma stem cells promotes self-renewal and M2-like polarization of tumor-associated macrophages

Yin et al.

Contents

Supplementary Figures 1-8

Supplementary Tables 1-3

SUPPLEMENTARY FIGURES 1-7

Supplementary Figure 1

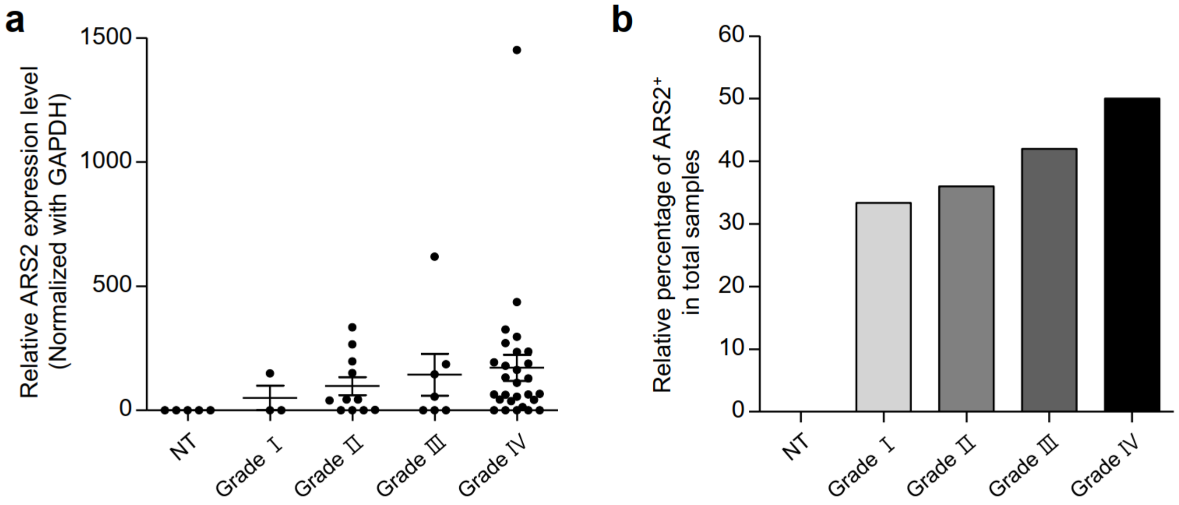

Supplementary Figure 1. Comparison of ARS2 expression level according to brain tumor grade. a, The dot plot of relative ARS2 expression level according to brain tumor grade in patient tissues from National Cancer Center (NCC), Republic of Korea. Expression level normalized by loading control GAPDH. (n; NT=5, Grade I=3, II=11, III=6, IV=26). All error bars represent mean ± standard error of the mean (SEM). **b,** The bar graph of ARS2 positive percentage according to brain tumor grade in patient tissues from NCC. The index score of y-axis are counted as percentages compared to total samples.

Supplementary Figure 2

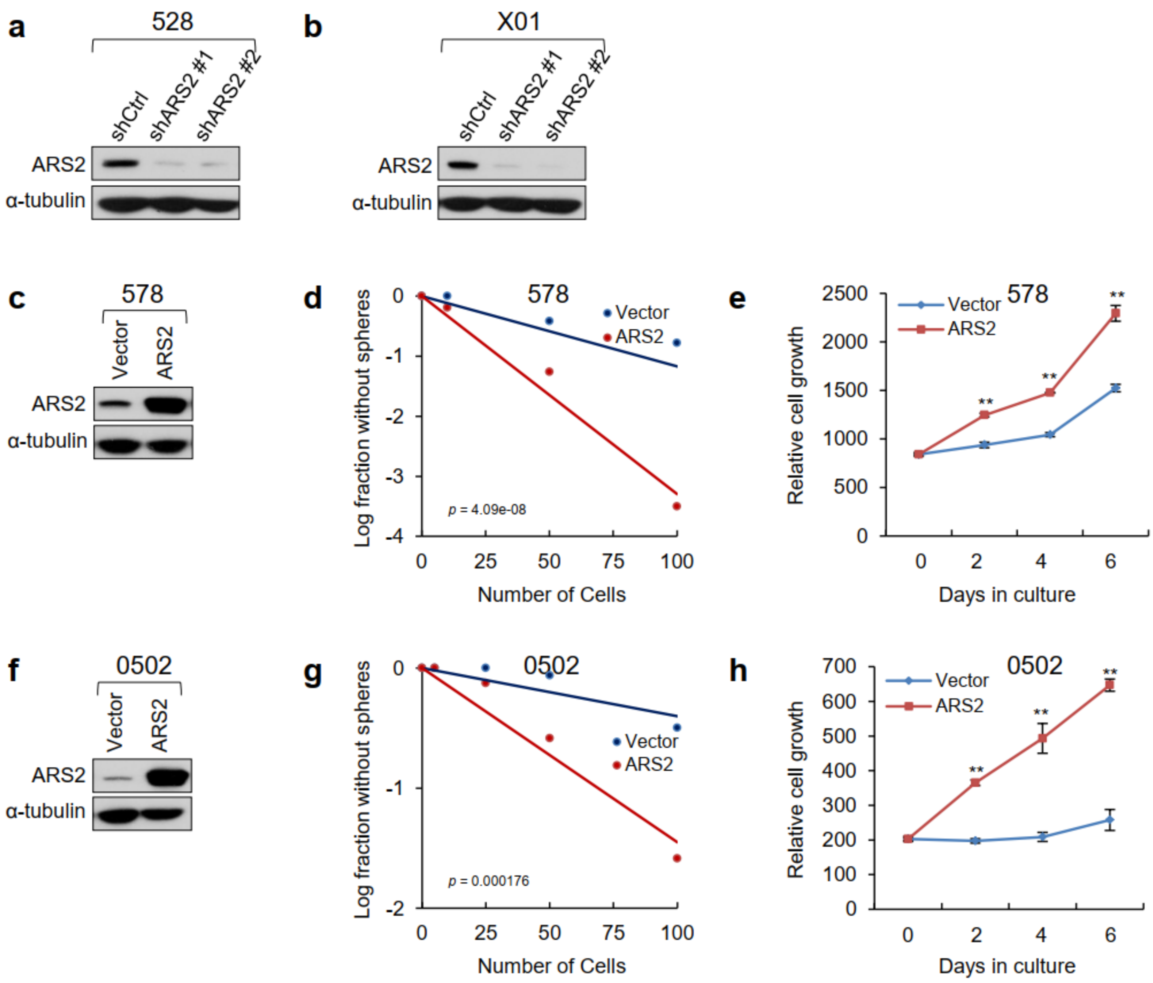

Supplementary Figure 2. Overexpression of ARS2 Promotes GSCs Self-renewal. a,b, Immunoblot (IB) analysis of ARS2 in GSCs (528 cells and X01 cells) infected with a shARS2-expressing lentiviral or shCtrl construct. α-tubulin was used as a loading control. **c,** IB analysis of ARS2 in GSCs (578 cells) infected with an ARS2-expressing lentiviral or vector construct. α-tubulin was used as a loading control. **d,** Limiting Dilution Assays (LDAs), performed in 578-Vector and 578- ARS2 cells. (n = 24, *t*-test). **e,** Cell proliferation assays performed using 578-Vector and 578- ARS2 cells. All error bars represent mean ± SEM (n=3). ** $P < 0.01$. **f,** IB analysis of ARS2 in GSCs (0502 cells) infected with an ARS2-expressing lentiviral or vector construct. α-tubulin was used as a loading control. **g,** LDAs, performed in 0502-Vector and 0502- ARS2 cells. (n = 24, *t*-test). **h,** Cell proliferation assays performed using 0502-Vector and 0502-ARS2 cells. All error bars represent mean ± SEM (n=3). ** $P < 0.01$.

Supplementary Figure 3

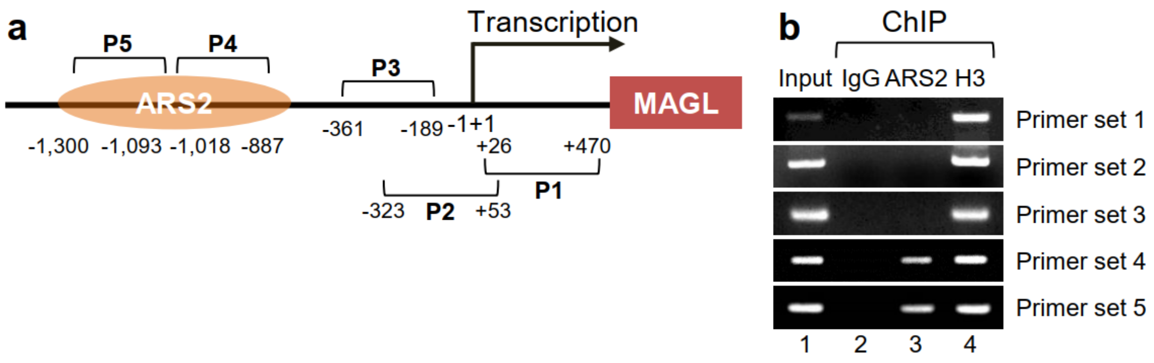

Supplementary Figure 3. ARS2 Directly Binding to MAGL Promoter. a, Schematic primer design for Chromatin immunoprecipitation (ChIP) analysis in MAGL promoter. **b,** Immunoprecipitated chromatin was analyzed by PCR using primer specific for the promoter region of the MAGL gene. An antibody against IgG was used as a nonspecific control and histone H3 antibody as positive ChIP grade control.

Supplementary Figure 4

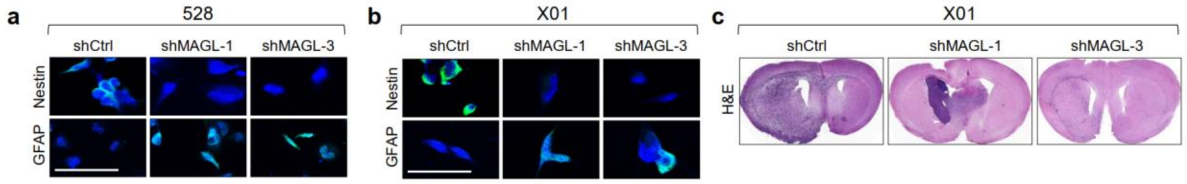

Supplementary Figure 4. MAGL Regulates GSCs Stemness. a,b, Representative immunocytochemistry (ICC) images of Nestin and GFAP in GSCs (528 cells and X01 cells) infected with a shMAGL-expressing lentiviral or shCtrl construct. Nuclei were counterstained with DAPI (blue). Scale bar, 50 μm. **c,** H&E staining of the whole brain in the orthotopic xenograft mouse model of GSCs (X01 cells) infected with a shMAGL-expressing lentiviral or shCtrl construct. The sample is extracted at 39 days after cell injection. Scale bar, 50 μm.

Supplementary Figure 5

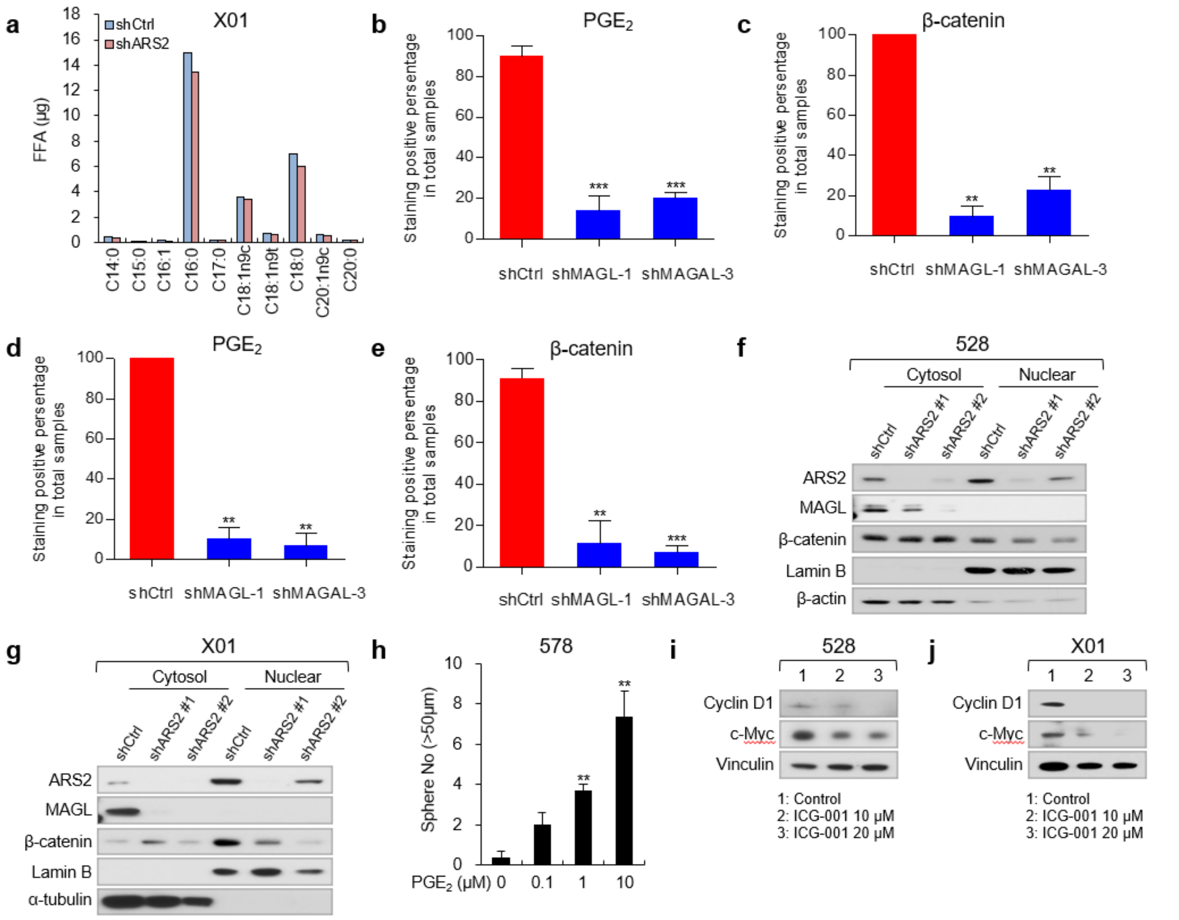

Supplementary Figure 5. MAGL Regulates Stemness of GSCs by PGE_2. a, Fatty Acid analysis performed using GSCs (X01 cells) infected with a shARS2-expressing lentiviral or shCtrl construct. (n = 2). **b,c,** The graph shows the average positive cells percentage of ICC analysis of PGE_2 and β-catenin in 528 cell infected with a shMAGL-expressing lentiviral or shCtrl construct. All error bars represent mean ± SEM (n=3). (** $P < 0.01$, *** $P < 0.001$, *t*-test). **d,e,** The graph shows the average positive cells percentage of ICC analysis of PGE_2 and β-catenin in X01 cell infected with a shMAGL-expressing lentiviral or shCtrl construct. All error bars represent mean ± SEM (n=3). (** $P < 0.01$, *** $P < 0.001$, *t*-test). **f,g,** Immunoblot (IB) analysis of ARS2, MAGL and β-catenin in fractionated nuclear or cytosolic lysates from 528 cells and X01 cells infected with a shARS2-expressing lentiviral or shCtrl construct. Lamin B and β-actin were used as markers for nucleus and cytoplasm, respectively. **h,** Sphere formation assay performed using GSCs (578 cells) treated with different concentrations of PGE_2 (0.1, 1, 10μM) or control. The graph shows the average number of spheres greater than

50 μm in diameter. All error bars represent mean ± SEM (n=3). (** $P < 0.01$, *t*-test). **i,j,** IB analysis of Cyclin D1 and c-Myc in GSCs (528 and X01 cells) treated with different concentrations of ICG-001 (10, 20μM) or control. Vinculin was used as a loading control.

Supplementary Figure 6

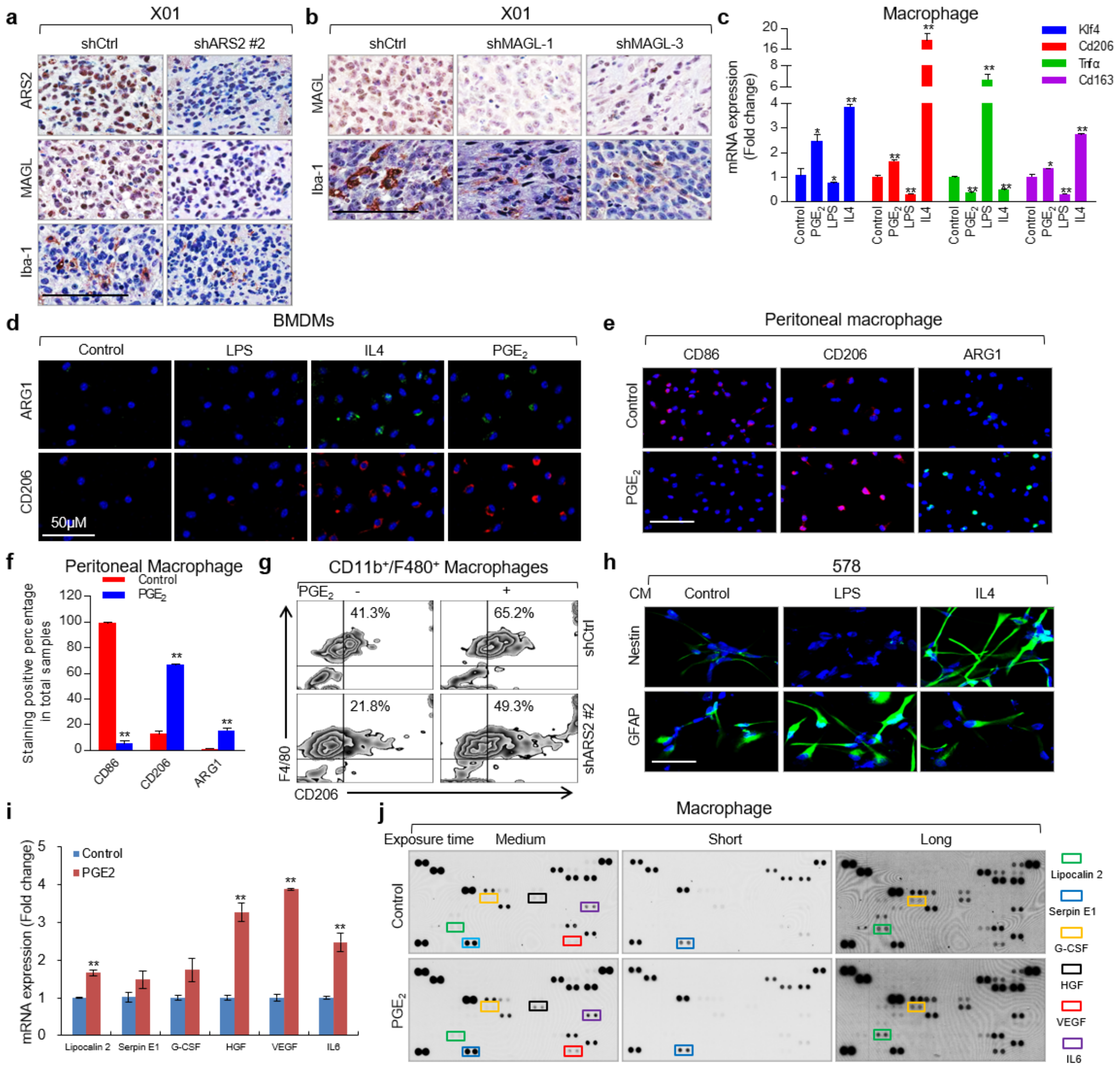

Supplementary Figure 6. ARS2/MAGL Expression Correlates with TAM Density. a, Representative immunohistochemistry (IHC) images of ARS2, MAGL, and Iba-1 in the orthotopic xenograft mouse model of GSCs (X01 cells) infected with a shARS2-expressing lentiviral or shCtrl construct. Scale bar, 100 μm. **b,** Representative IHC images of MAGL and Iba-1 in the orthotopic xenograft mouse model of GSCs (X01 cells) infected with a shMAGL-expressing lentiviral or shCtrl construct. Scale bar, 100 μm. **c,** qRT-PCR analysis of Klf4, Cd206, Tnfα, and Cd163 in peritoneal mouse macrophages after induction with PGE_2 (10 μM), LPS or IL4, or treatment with control. All error bars represent mean ± SEM (n=3). (* $P < 0.05$, ** $P < 0.01$, *t*-test). **d,** Representative ICC images of ARG1 and CD206 in bone marrow derived-macrophages (BMDMs) after treatment with LPS, IL-4, PGE_2 (10 μM),

or control. Nuclei were counterstained with DAPI (blue). Scale bar, 50 μm. **e, f,** Representative ICC images (e) and corresponding quantification (f) of CD86, CD206, and ARG1 in peritoneal mouse macrophages after treatment with PGE_2 (10 μM) or control. Scale bar, 50 μm. All error bars represent mean ± SEM (n=3). (** $P < 0.01$, *t*-test). **g,** Expression of CD206 in macrophages treated with PGE_2 or vehicle control for 24hrs by flow cytometry. CD11b and F4/80 was co-stained for macrophagic population verification. **h,** Representative ICC images of Nestin and GFAP in GSCs (578 cells) cultured in conditioned media (CM) from peritoneal mouse macrophages after induction with LPS or IL4, or treatment with control. Nuclei were counterstained with DAPI (blue). Scale bar, 50 μm. **i,** qRT-PCR analysis of Lipocalin 2, Serpin E1, G-CSF, HGF, VEGF and IL6 in peritoneal mouse macrophages after induction with PGE_2 (10 μM). All error bars represent mean ± SEM (n=3). (** $P < 0.01$, *t*-test). **j,** Cytokine array performed using peritoneal mouse macrophages after induction with PGE_2 (10 μM).

Supplementary Figure 7

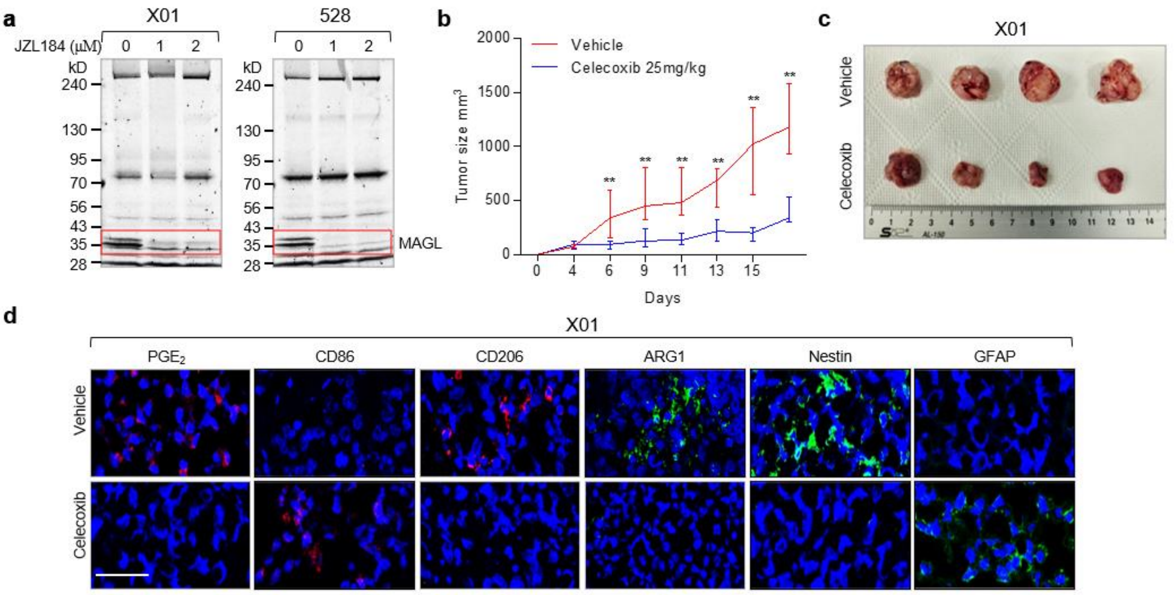

Supplementary Figure 7. Celecoxib inhibits GSC self-renewal and tumorigenicity. a, Immunoblot (IB) analysis of MAGL hydrolase activity in GSCs (X01, 528 cells) treated with JZL184 (0, 1, 2 μM). **b,** Comparative analysis of subcutaneous tumor size of mice implanted with X01 treated with Celecoxib 25mg/kg or vehicle. All error bars in the graph represent mean ± SD. (n=4, $3X10^6$ cells injected per mouse). ** $P < 0.01$, *t*-test. **c,** Images of tumor tissues from mice treated with vehicle or celecoxib. **d,** Representative IF images of PGE_2, CD86, CD206, ARG1, Nestin and GFAP in a celecoxib-treated subcutaneous mouse model. Sale bar, 50μM.

Supplementary Figure 8

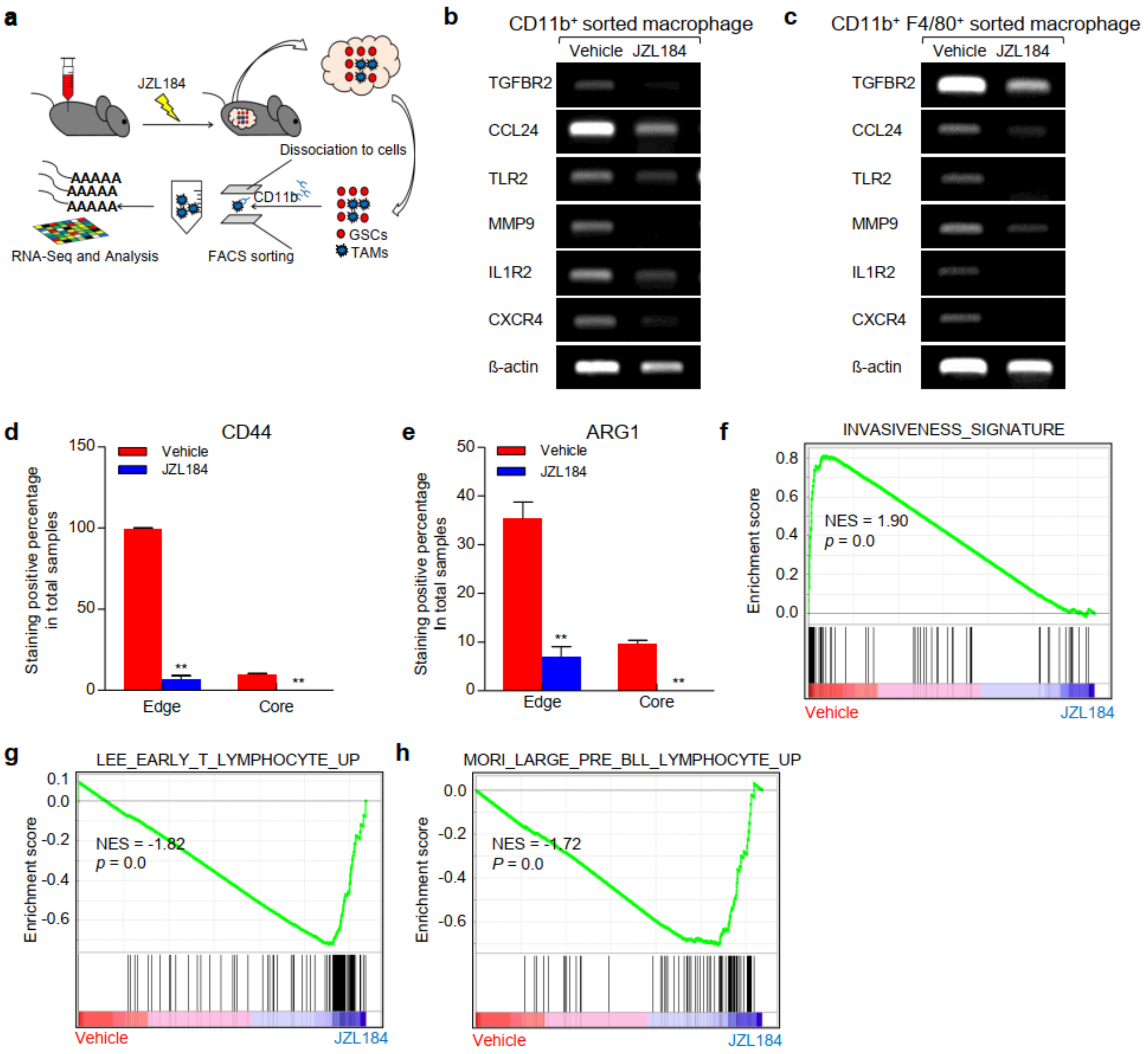

Supplementary Figure 8. JZL184 downregulates M2-like TAM markers expression. a, Schematic representation of the fluorescence-activated cell sorting (FACS) of CD11b$^+$ macrophages from the subcutaneous mouse models of GCS X01. **b,c,** RT-PCR analysis of M2-like TAMs markers in CD11b$^+$ (b) and CD11b$^+$/F4/80$^+$ (c) sorted macrophages treated with JZL184 or vehicle. ß-actin was used as a loading control. **d,e,** The graph shows the staining positive percentage of IHC and IF analysis of CD44 (d), ARG1 (e) at region of tumor edge or core of brain slices treated with JZL184 or vehicle. All error bars represent mean ± SEM (n=3). (n = 3, ** $P < 0.01$, *t*-test). **f** Gene set enrichment assay (GSEA) plot for invasiveness gene signature in comparison of TAMs from vehicle- vs. JZL184-treated subcutaneous mouse model. **g,h,** GSEA plot for two types of lymphocyte gene signature in comparison of TAMs from vehicle- vs. JZL184-treated subcutaneous mouse model.

SUPPLEMENTARY TABLES 1-3

Supplementary Table 1. Oligonucleotides used in PCR-based cloning of various plasmid constructs.

* S, sense; A, antisense

Plasmid constructs	Sequence of oligonucleotides
pcDNA3-ARS2-FLAG	S: 5′- CGG GAT CCG CCA CCA TGG GTG ACA GTG ATG ACG -3′<br>A: 5′- GCT CTA GAA AGA AAT CAA CAT CGT CTG GG -3′
pLenti6-ARS2-FLAG	S: 5′- AGT GTG GTG GAA TTC GGA TCC GCC ACC ATG GG -3′<br>A: 5′- CCC TCT AGA CTC GAG GGC CCT ACT TGT CAT CGT CG -3′
pLenti6-MAGL	S: 5′- CGG GAT CCG CCA CCA TGC CAG AGG AAA GTT CCC -3′<br>A: 5′- GGA ATT CAC CGG CCA ATG CAT TCA G -3′
pGreen-MAGL-pro.	S: 5′- TTT TAT CGA TGA ATT CCG GTG CAC TTA GCA TGT C -3'<br>A: 5′- TAC ACG CCT AAC TAG TCA TCG GAA ATG CCG CTG G-3'

Supplementary Table 2. Oligonucleotides used in shRNA expressing lentivirus constructs.

* S, sense; A, antisense

Plasmid constructs	Sequence of oligonucleotides
pLKO.1puro-shARS2-#1	S: 5′- CCG GGC TGA GAA TGA CAG TTC TAA TCT CGA GAT TAG AAC TGT CAT TCT CAG CTT TTT G -3′<br>A: 5′- AAT TCA AAA AGC TGA GAA TGA CAG TTC TAA TCT CGA GAT TAG AAC TGT CAT TCT CAG C -3′
pLKO.1puro-shARS2-#2	S: 5′- CCG GGC CAT TGT CAA GAT GCT GGA TCT CGA GAT CCA GCA TCT TGA CAA TGG CTT TTT G -3′<br>A: 5′- AAT TCA AAA AGC CAT TGT CAA GAT GCT GGA TCT CGA GAT CCA GCA TCT TGA CAA TGG-3′
pLKO.1puro-shMGLL-#1	S: 5′- CCG GCA ACT CCG TCT TCC ATG AAA TCT CGAGAT TTC ATG GAA GAC GGA GTT GTT TTT G-3'<br>A: 5′- AAT TCA AAA ACA ACT CCG TCT TCC ATG AAA TCT CGA GAT TTC ATG GAA GAC GGA GTT G -3'
pLKO.1puro-shMGLL-#2	S: 5′- CCG GCC AGG ACA AGA CTC TCA AGA TCT CGA GAT CTT GAG AGT CTT GTC CTG GTT TTT G-3'<br>A: 5′- AAT TCA AAA ACC AGG ACA AGA CTC TCA AGA TCT CGA GAT CTT GAG AGT CTT GTC CTG G -3'

Supplementary Table 3. Oligonucleotides used in ChIP and semi-quantitative PCR analysis.

* S, sense; A, antisense

Genes	Sequence of oligonucleotides
Primer set 1	S: 5′- CCGGCCCAGGGATAAAGTGG -3′<br>A: 5′- TCGGAAATGCCGCTGGGAAA -3′
Primer set 2	S: 5′-TACACGTGTGGTGAGTGTGC-3′<br>A: 5′-CTGCGCCGCCACTTTATC-3′
Primer set 3	S: 5′-CCTGCGTGCAGTGTAGTGAC-3′<br>A: 5′-GAACTGAGCTGGGTTCATGG-3′
Primer set 4	S: 5′- AGC GGA GGA GCT AAT GTT CA -3′<br>A: 5′- GCT GTG ACC CCC AGA TAA AA -3′
Primer set 5	S: 5′- CAG TGG GTA AGT CAC GCT CA -3′<br>A: 5′- CCA GTG GAG TGT CCC TGT CT -3′

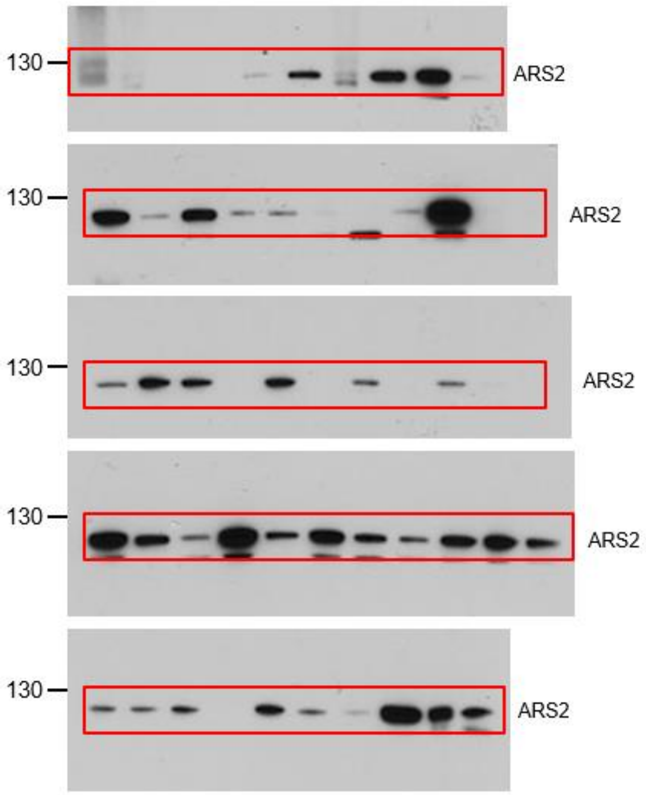

Original immunoblot for Fig. 1f.

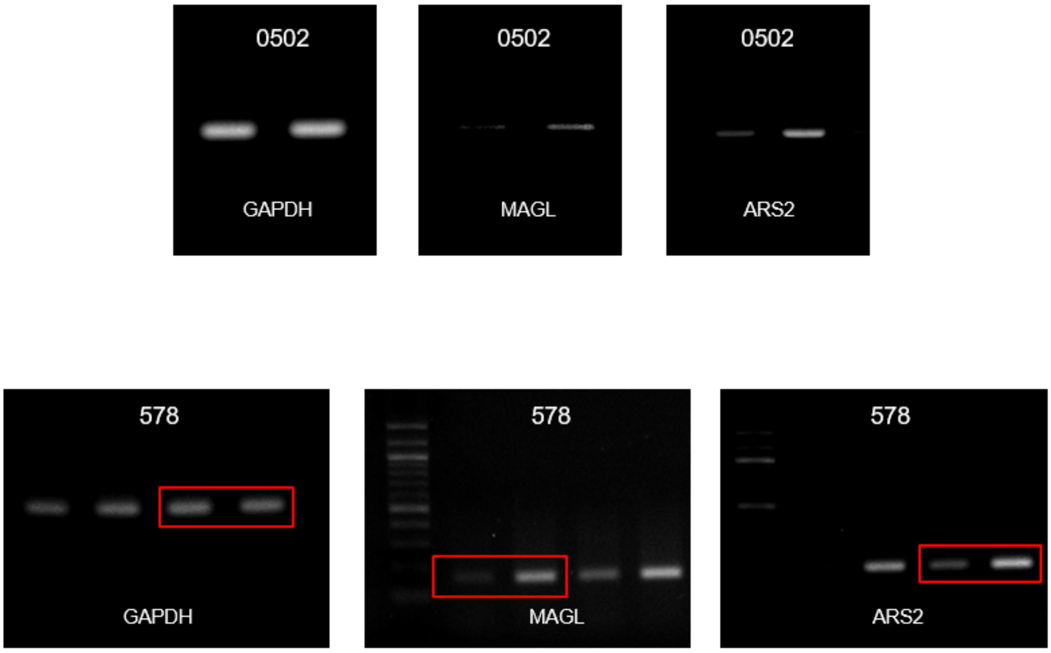

Original RT-PCR for Fig. 3b,c.

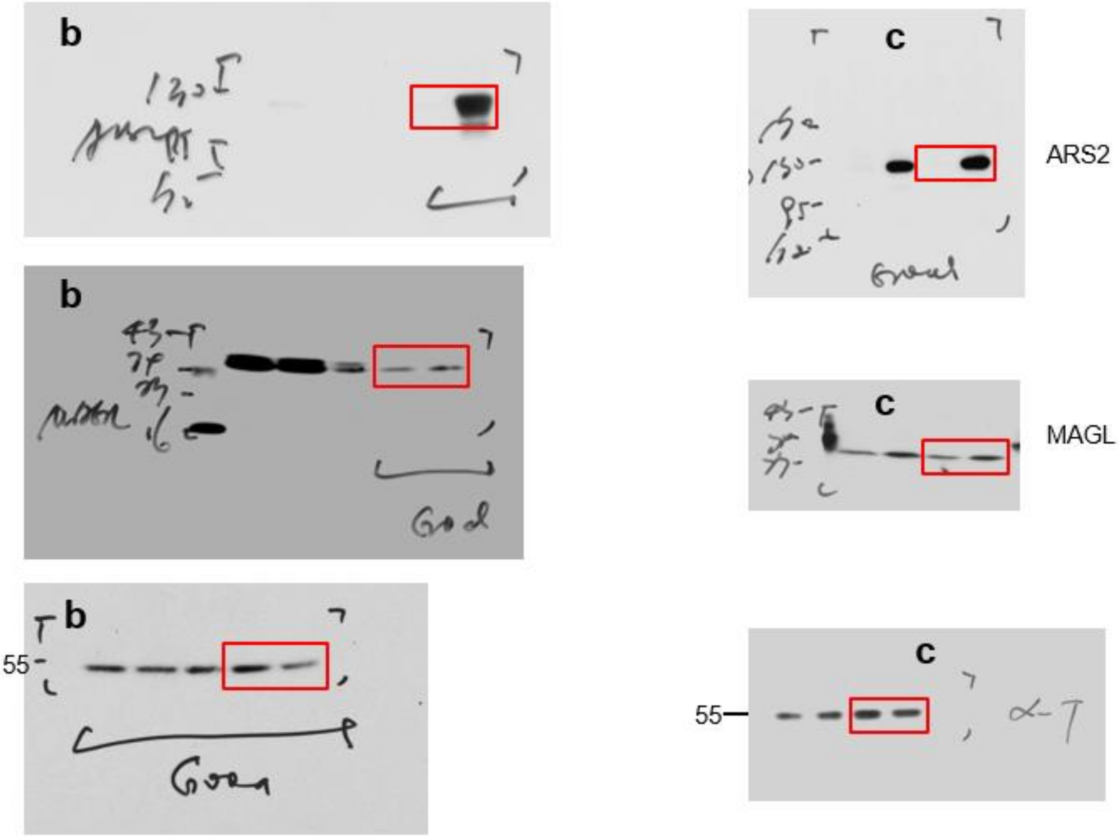

Original immunoblot for Fig. 3b,c.

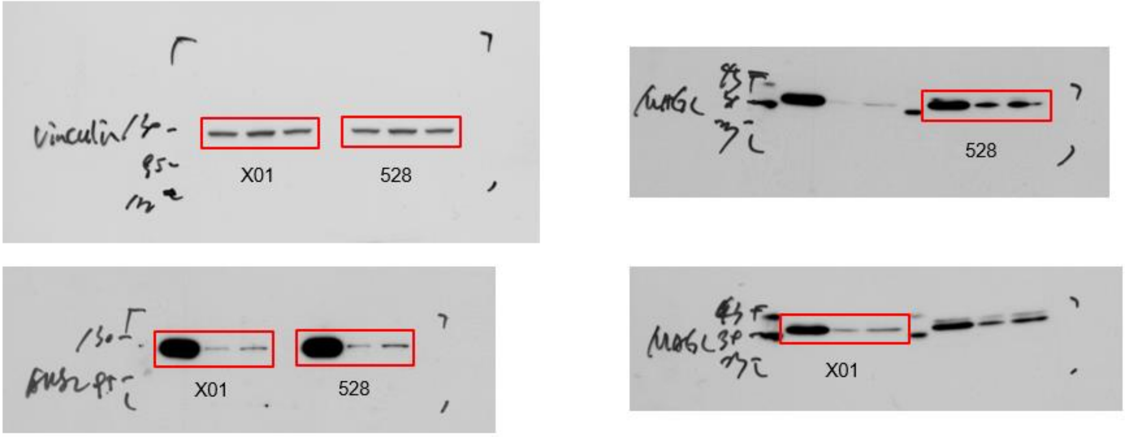

Original immunoblot for Fig. 3d,e.

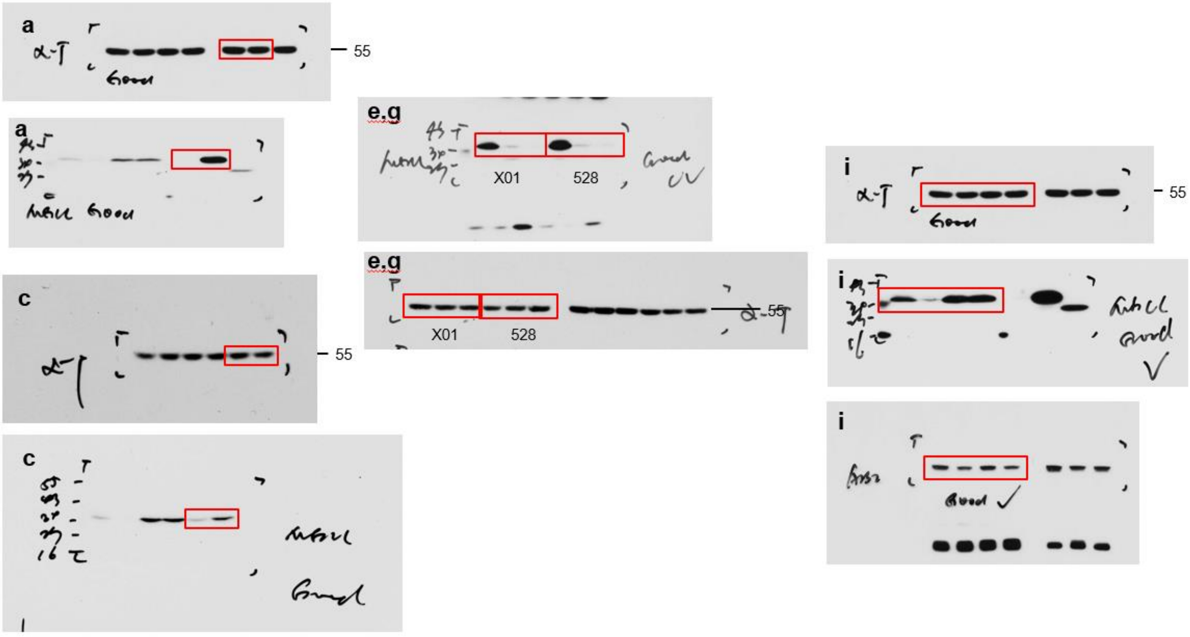

Original immunoblot for Fig. 4a,c,e,g,i.

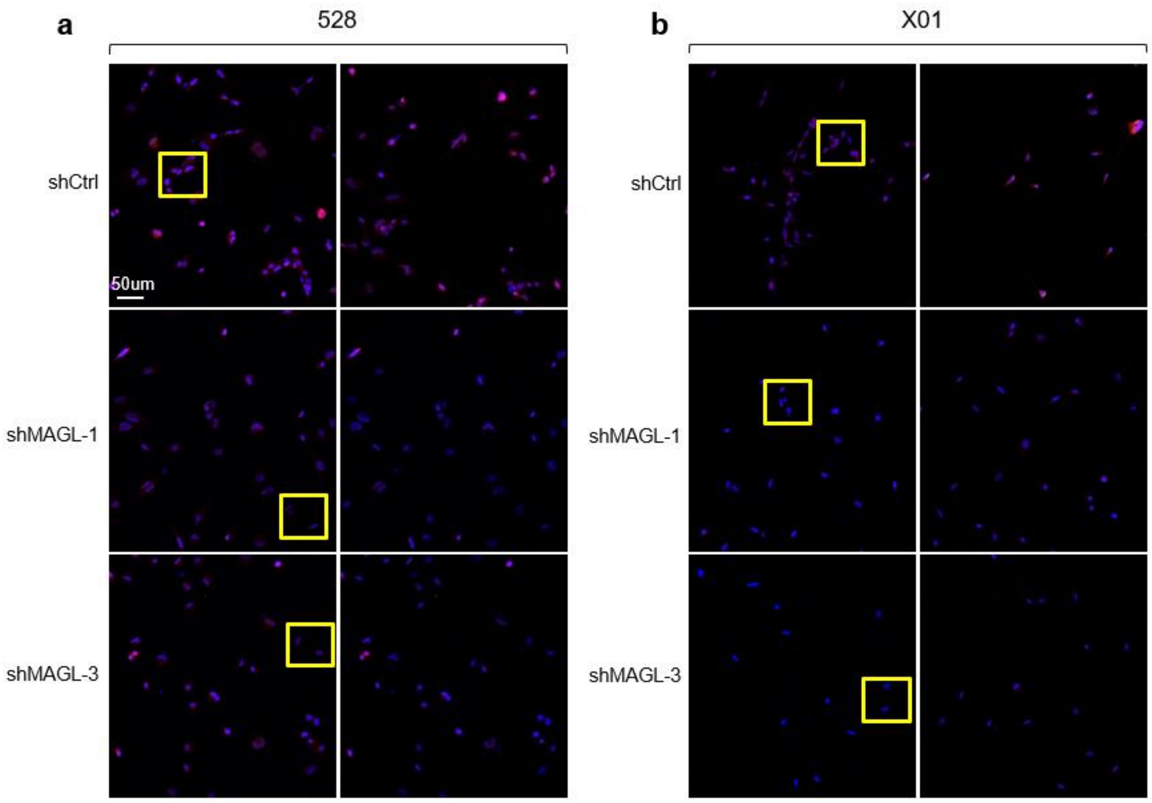

Original ICC staining (PGE_2) for Fig. 5a,b.

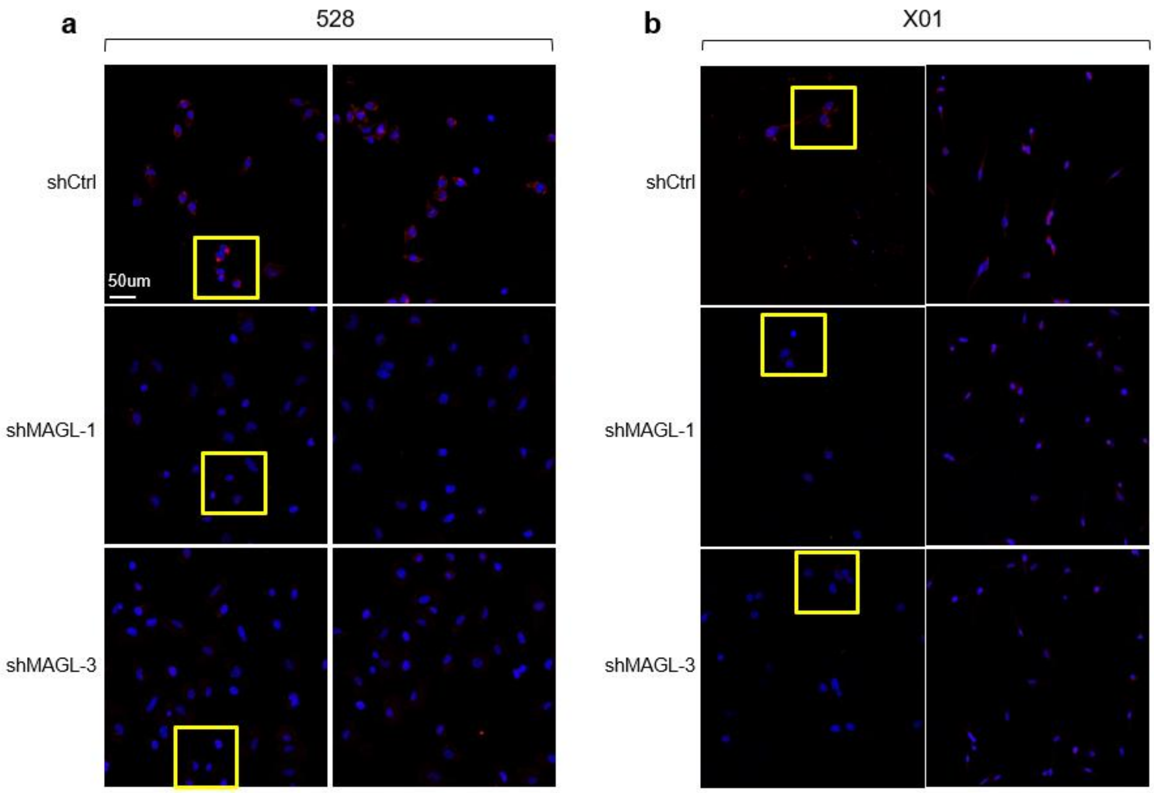

Original ICC staining (β-Catenin) for Fig. 5a,b.

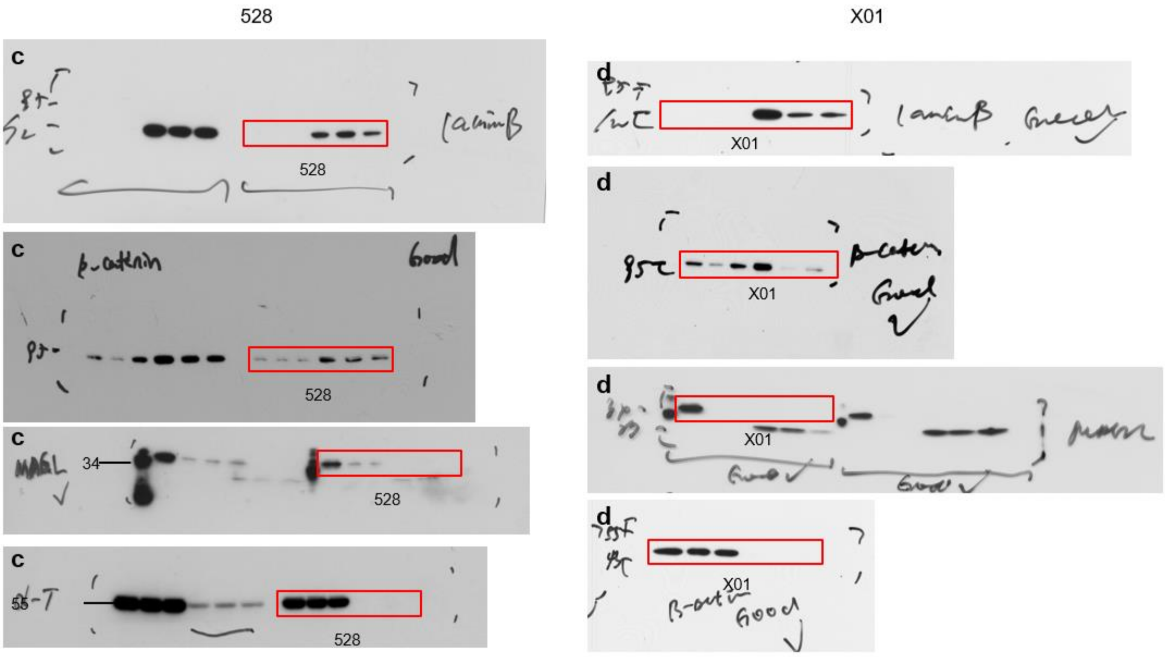

Original immunoblot for Fig. 5c,d.

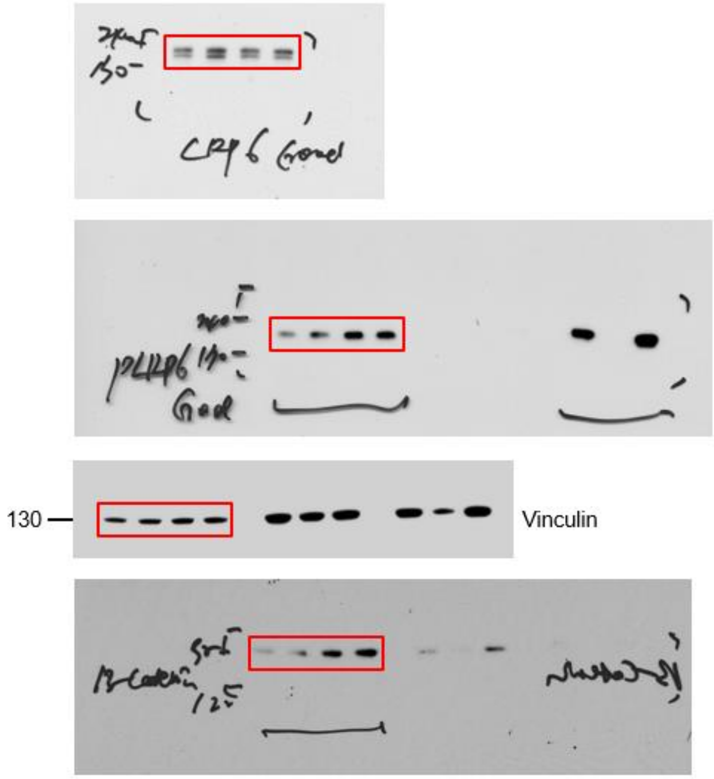

Original immunoblot for Fig. 5e.

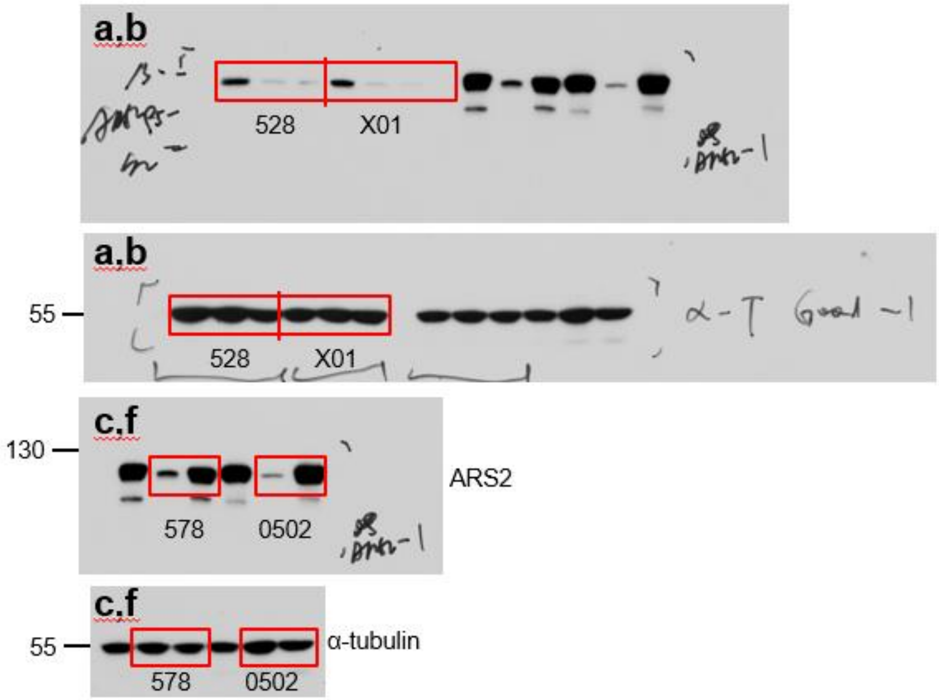

Original immunoblot for Supplementary Fig. 2a,b,c,f.

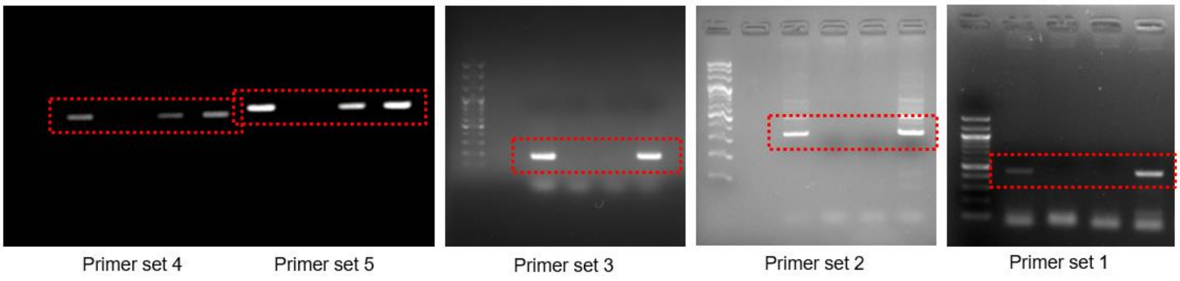

Original RT-PCR for Supplementary Fig. 3b.

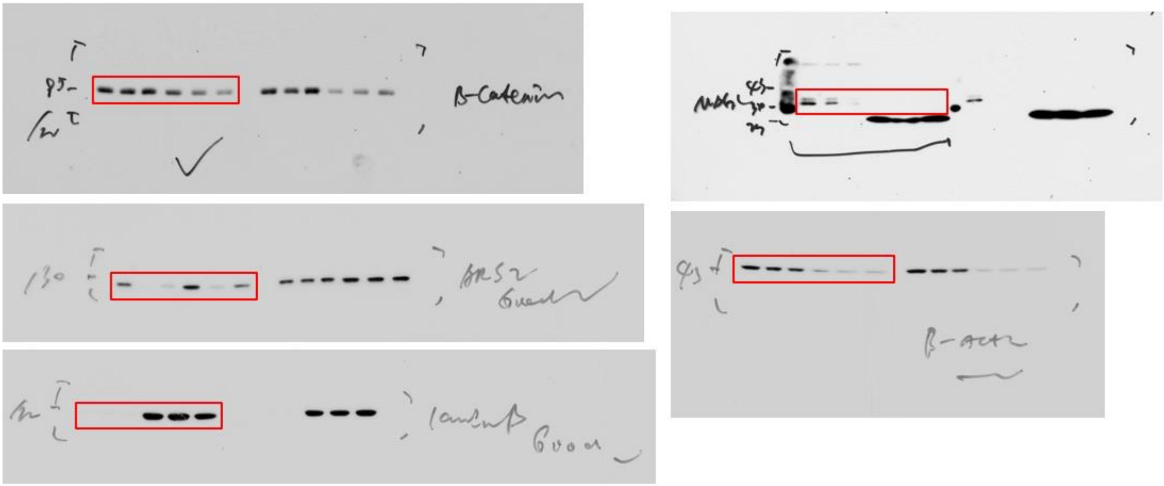

Original immunoblot for Supplementary Fig. 5f.

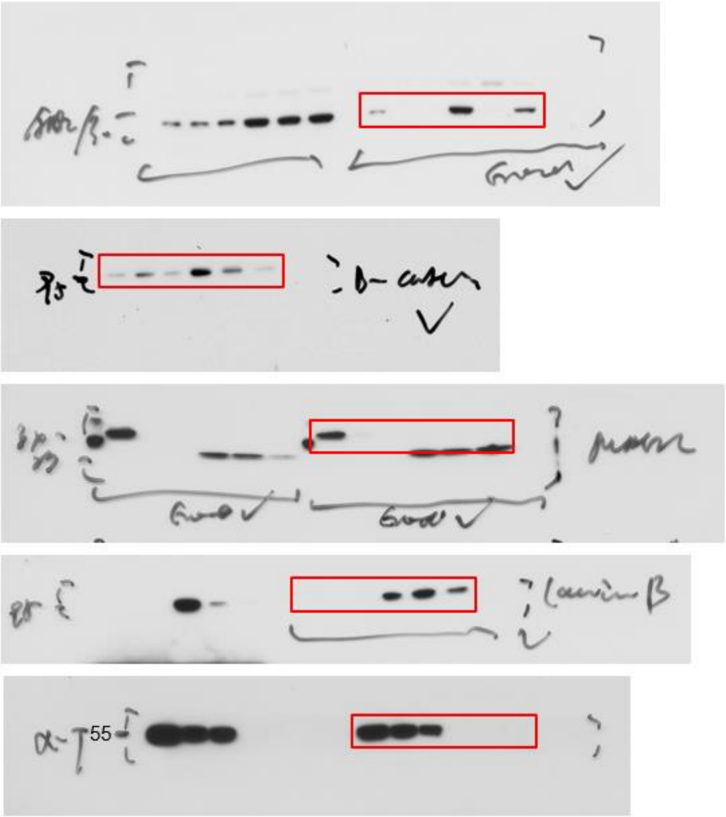

Original immunoblot for Supplementary Fig. 5g.

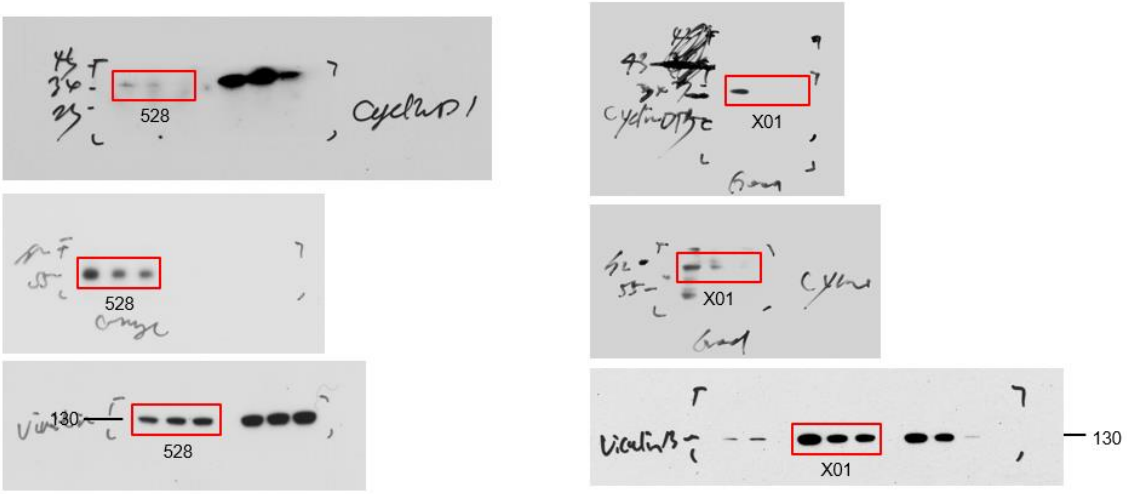

Original immunoblot for Supplementary Fig. 5i,j.

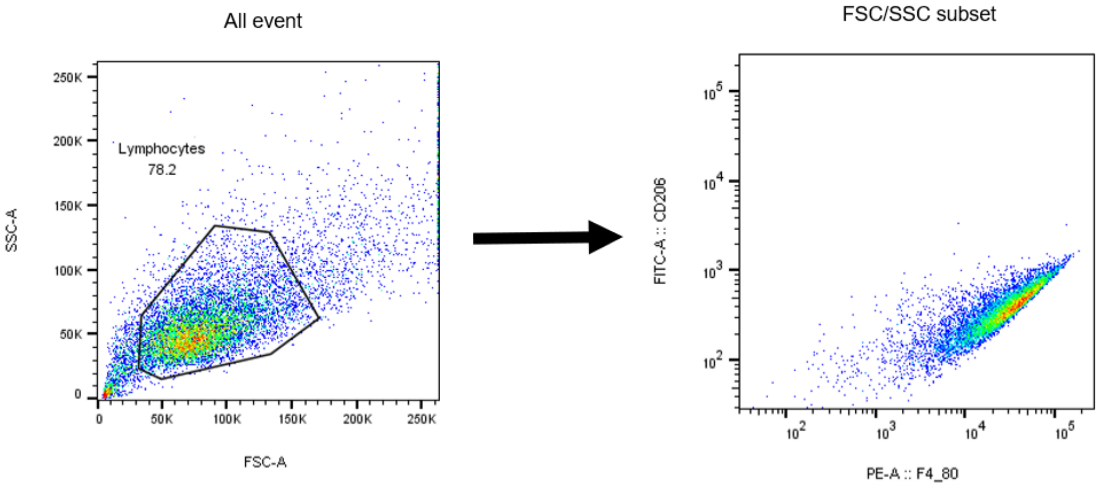

Original FACS gating method for Supplementary Fig. 6c,d.

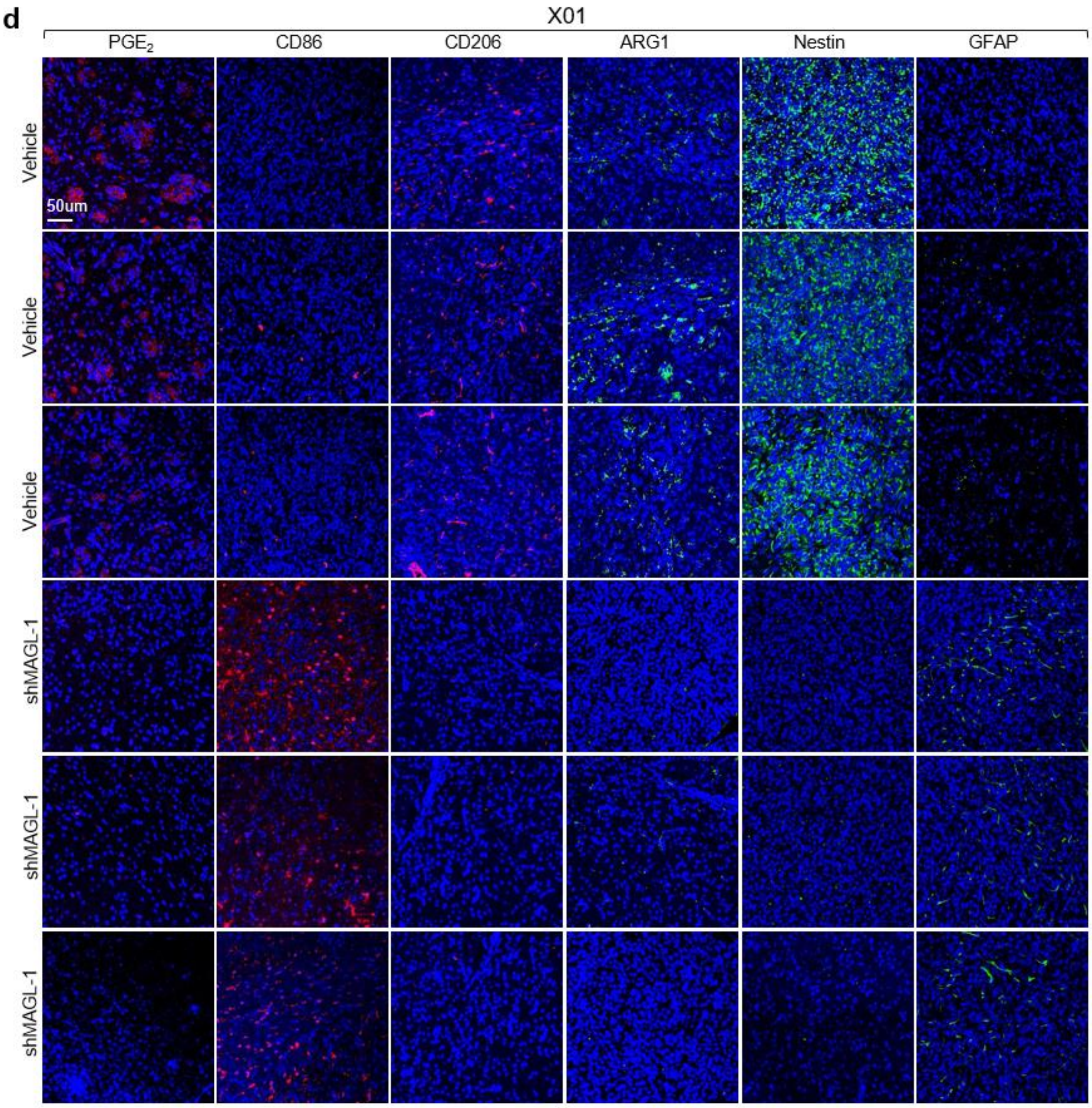

1
2 **Original ICC staining for Fig. 6d.**

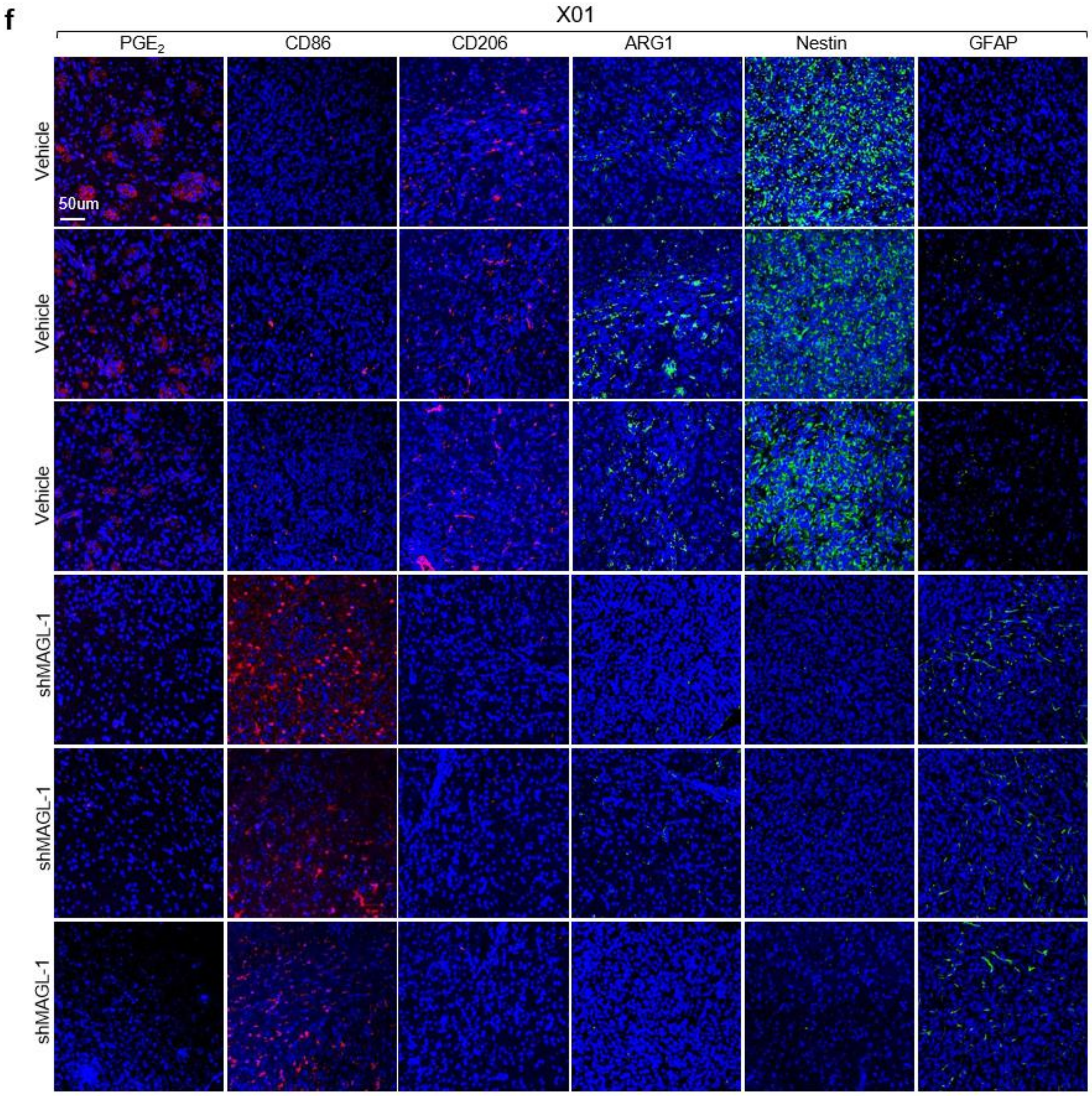

Original ICC staining for Fig. 6f.

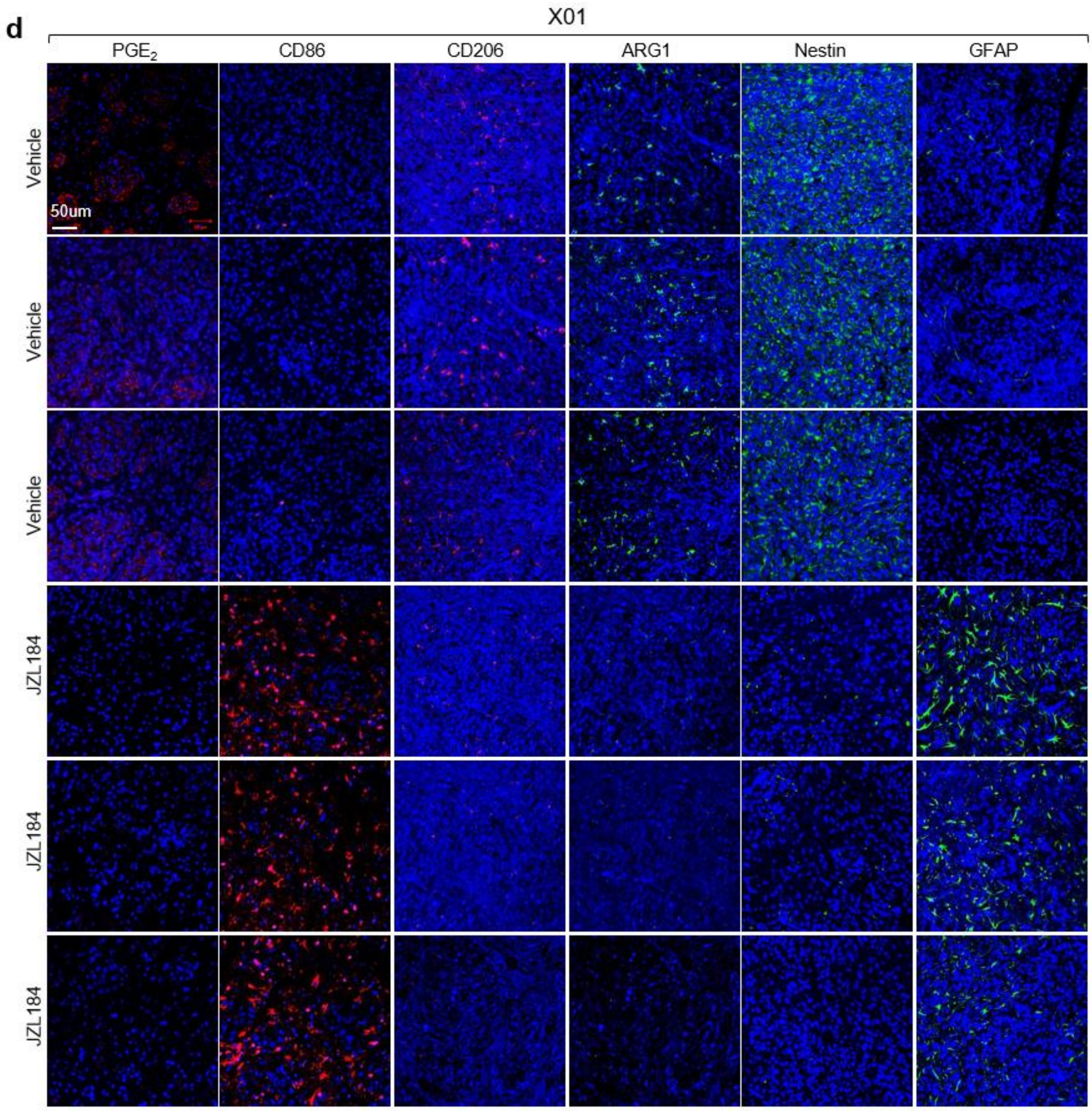

Original ICC staining for Fig. 7d.

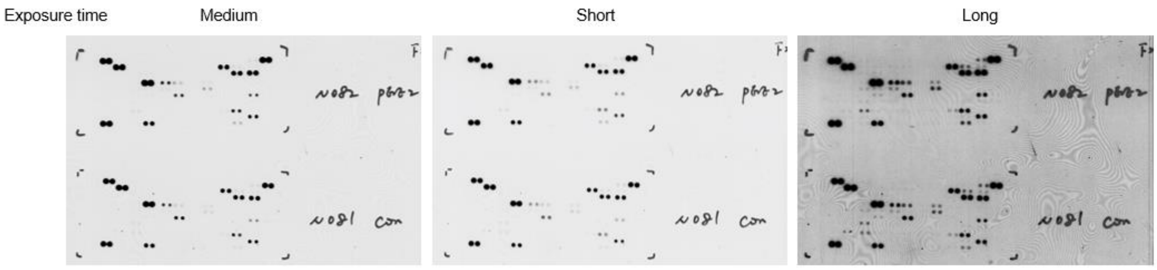

Original image for Supplementary Fig. 6i .

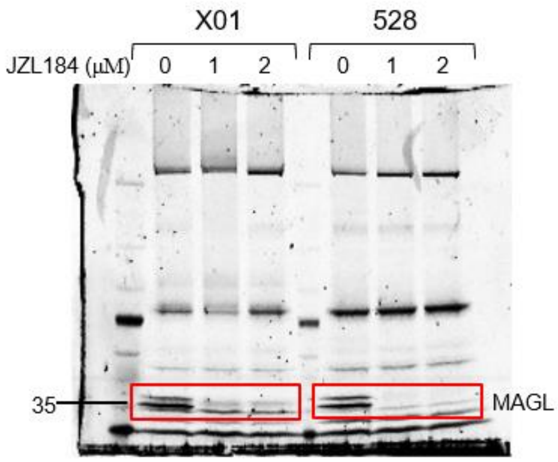

Original gel image for Supplementary Fig. 7a.

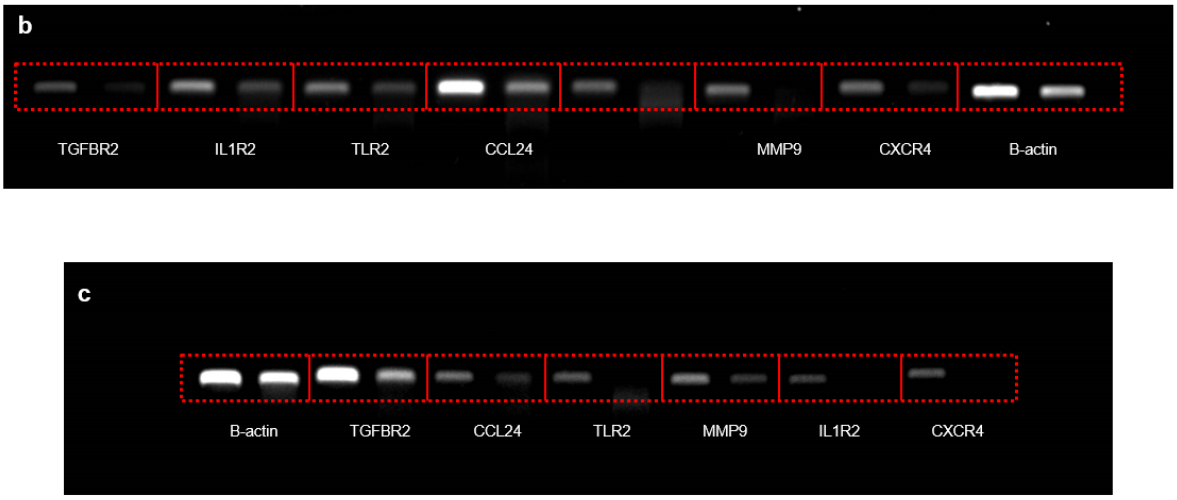

Original RT-PCR for Supplementary Fig. 8b, c.
