# Supplementary material for: ARS2/MAGL signaling in glioblastoma stem cells promotes self-renewal and M2-like polarization of tumor-associated macrophages
Source: Nat Commun. 2020 Jun 12;11:2978. doi: 10.1038/s41467-020-16789-2 (PMC7293269; doi:10.1038/s41467-020-16789-2)
Supplement: Supplementary file 3 — Reporting Summary [file 41467_2020_16789_MOESM3_ESM.pdf]

nature research

Corresponding author(s): Jong Bae Park

Last updated by author(s): May 15, 2020

Reporting Summary

Nature Research wishes to improve the reproducibility of the work that we publish. This form provides structure for consistency and transparency in reporting. For further information on Nature Research policies, see our Editorial Policies and the Editorial Policy Checklist.

Statistics

For all statistical analyses, confirm that the following items are present in the figure legend, table legend, main text, or Methods section.

n/a	Confirmed	
☐	☒	The exact sample size (*n*) for each experimental group/condition, given as a discrete number and unit of measurement
☐	☒	A statement on whether measurements were taken from distinct samples or whether the same sample was measured repeatedly
☐	☒	The statistical test(s) used AND whether they are one- or two-sided<br>*Only common tests should be described solely by name; describe more complex techniques in the Methods section.*
☐	☒	A description of all covariates tested
☐	☒	A description of any assumptions or corrections, such as tests of normality and adjustment for multiple comparisons
☐	☒	A full description of the statistical parameters including central tendency (e.g. means) or other basic estimates (e.g. regression coefficient) AND variation (e.g. standard deviation) or associated estimates of uncertainty (e.g. confidence intervals)
☐	☒	For null hypothesis testing, the test statistic (e.g. *F*, *t*, *r*) with confidence intervals, effect sizes, degrees of freedom and *P* value noted<br>*Give P values as exact values whenever suitable.*
☒	☐	For Bayesian analysis, information on the choice of priors and Markov chain Monte Carlo settings
☐	☒	For hierarchical and complex designs, identification of the appropriate level for tests and full reporting of outcomes
☐	☒	Estimates of effect sizes (e.g. Cohen's *d*, Pearson's *r*), indicating how they were calculated

Our web collection on statistics for biologists contains articles on many of the points above.

Software and code

Policy information about availability of computer code

Data collection	No software was used for data collection.
Data analysis	ELDA software (ver 2014), Microsoft Excel (ver 2010), GraphPad Prism (ver 5 and ver 7), R (ver 3.2.1), ssGSEA2-2.2.1 (ver 2.2.1), Subread aligners (ver 1.5.3), MeV (ver 4.9.0), SeqMonk software (ver 1.38.2), FlowJo software (ver 10.6.2), HISAT (ver 2.0.5).

For manuscripts utilizing custom algorithms or software that are central to the research but not yet described in published literature, software must be made available to editors and reviewers. We strongly encourage code deposition in a community repository (e.g. GitHub). See the Nature Research guidelines for submitting code & software for further information.

Data

Policy information about availability of data

All manuscripts must include a data availability statement. This statement should provide the following information, where applicable:
- Accession codes, unique identifiers, or web links for publicly available datasets
- A list of figures that have associated raw data
- A description of any restrictions on data availability

All ARS2 (SRRT) bioinformatic data was collected from REMBRANDT (currently hosted by GDoC), TCGA data portal (http://cancergenome.nih.gov/) and the Cancer Genomics Hub (http:/cghub.ucsc.edu). RNA sequencing data have been uploaded in European Genome-phenome Archive (EGA) with EGA-box-1261 accession code and NCBI Gene Expression Omnibus (GEO) with GSE150630 and GSE150631. Accession codes for all datasets will be available without any restriction following Nature policy.
The list of figures that have associated biological raw data are:
Figure 1 h;
Figure 2 b, c, e, f, h;

Figure 3 d, e, f, g, h;
Figure 4 b, d, f, h, j, k;
Figure 5 g, h, i;
Figure 6 a, e, g;
Figure 7 a, b, e, f;
Figure 8 f;
Supplementary Figure 1 a, b;
Supplementary Figure 2 d, e, g, h;
Supplementary Figure 5 a, b, c, d, e, h;
Supplementary Figure 6 c, f, i;
Supplementary Figure 7 b;
Supplementary Figure 8 d, e;

Field-specific reporting

Please select the one below that is the best fit for your research. If you are not sure, read the appropriate sections before making your selection.

☒ Life sciences ☐ Behavioural & social sciences ☐ Ecological, evolutionary & environmental sciences

For a reference copy of the document with all sections, see nature.com/documents/nr-reporting-summary-flat.pdf

Life sciences study design

All studies must disclose on these points even when the disclosure is negative.

Sample size	No statistical methods were used to predetermine sample size. All available samples passing the quality control were included.
Data exclusions	No data were excluded from the analyses.
Replication	Three technical replicates were performed and experiments were repeated at least three times with similar results. All attempts at replication were successful.
Randomization	No experimental grouping requiring randomization was performed.
Blinding	The investigators and authors have been consistently blinded to the group allocation during data collection and/or analysis.

Reporting for specific materials, systems and methods

We require information from authors about some types of materials, experimental systems and methods used in many studies. Here, indicate whether each material, system or method listed is relevant to your study. If you are not sure if a list item applies to your research, read the appropriate section before selecting a response.

Materials & experimental systems

n/a	Involved in the study	
☐	☒	Antibodies
☐	☒	Eukaryotic cell lines
☒	☐	Palaeontology and archaeology
☐	☒	Animals and other organisms
☐	☒	Human research participants
☒	☐	Clinical data
☒	☐	Dual use research of concern

Methods

n/a	Involved in the study	
☒	☐	ChIP-seq
☐	☒	Flow cytometry
☒	☐	MRI-based neuroimaging

Antibodies

Antibodies used

Immunoblot analysis: ARS2 (GeneTex, GTX119872, 1:500), MAGL (Santa Cruz, ab24701, 1:1000), pLRP6 (Ser1490, Cell Signaling, #2568, 1:1000), LRP6 (C47E12, Cell Signaling, #3395, 1:1000), β-Catenin (D10A8, Cell signaling, #8480, 1:500), Lamin B (C-20, Santa Cruz, sc-6216, 1:1000), Cyclin D1 (Santa Cruz Biotech, A-12, 1:1000), c-Myc (Santa Cruz Biotech, C-12, 1:1000), α-tubulin (TU-02, Santa Cruz, sc-8035, 1:1000), β-actin (C4, Santa Cruz, sc-47778, 1:1000), Vinculin (Sigma-Aldrich, V4139, 1:1000).
Chromatin immunoprecipitation: IgG (MAGnify Chromatin Immunoprecipitation System, Thermo-Fisher Scientific), histone H3 (Abcam, ab18521, 1:1000).
Immunocytochemical staining: Nestin (BD Biosciences, 611658, 1:500), GFAP (MP Biomedicals, 691102, 1:500), ARS2 (Genetex, GTX119872, 1:500), β-Catenin (D10A8, Cell signaling, #848, 1:500), CD86 (EP1158Y, Abcam, ab53004, 1:500), CD206 (Abcam, ab64693, 1:500), Arginase-1 (E-2, Santa Cruz, sc-271430, 1:500), PGE2 (Abcam, ab2318, 1:100).
Histology and tissue staining: Iba-1 (Wako, 019-19741, 1:500), CD44 (146-3C11, Cell Signaling, #3570, 1:500), ARS2 (GeneTex,

GTX119872, 1:500), MAGL (clone 1B1, LSbio, LS-C173047, 1:500), Nestin (BD Biosciences, 611658, 1:500), GFAP (MP Biomedicals, 691102, 1:500), PGE2 (Abcam, ab2318, 1:100), CD86 (EP1158Y, Abcam, ab53004, 1:500), CD206 (Abcam, ab64693, 1:500), Arginase-1 (E-2, Santa Cruz, sc-271430, 1:500).
FACs: anti-mouse F4/80 (PE, Invitrogen, 1:100), CD11b (PerCP-Cyanine5.5, Invitrogen, 1:100), CD86 (APC, BioLegend, 1:100), anti-mouse CD206 (FITC, BioLegend, 1:100).

Validation	Antibodies used were commercially available and were validated in multiple previous studies.

Eukaryotic cell lines

Policy information about cell lines

Cell line source(s)	GSC X01 and 0502 (Generous gift from Myung-Jin Park, Korea Institute of Radiological & Medical Sciences, South Korea)<br>GSC 578 (Generous gift from Do-Hyun Nam, Samsung Medical Center, South Korea)<br>GSC 528 (Generous gift from Ichiro Nakano, University of Alabama at Birmingham, United States)<br>GL261 (Generous gift from Jeongwu Lee, Lerner Research Institute, United States)
Authentication	We authenticated all GSCs by short tandem repeat (STR) analysis.
Mycoplasma contamination	We used Universal Mycoplasma Detection Kit to verify absence of mycoplasma contamination in our cell line. There were found to be negative.
Commonly misidentified lines<br>(See ICLAC register)	No commonly misidentified cell lines were used in this study.

Animals and other organisms

Policy information about studies involving animals; ARRIVE guidelines recommended for reporting animal research

Laboratory animals	Mouse, BalbC/nude, C57BL/6, Female, 5 weeks<br>Temperature and humidity are set to 22±1خ and 50±20%, respectively, in terms of range and are recorded every ten minutes. A sensor is installed in each animal room. If the temperature exceeds 25خ ,the designated individuals are notified via mobile phone by SMS. A total of four cooling units, four air handling units, and a central heating system are operated at the temperature and humidity that were automatically set. The operation is monitored 24 hours a day by a central monitoring system.
Wild animals	No wild animals are used in this research.
Field-collected samples	No field-collected samples are used in this research.
Ethics oversight	This animal studies were approved by National Cancer Center, South Korea of animal experiments ethics committees. The document number is 18-NCCIBC-009. Our institute was reviewed and approved by the Institutional Animal Care and Use Committee (IACUC) of National Cancer Center Research Institute.

Note that full information on the approval of the study protocol must also be provided in the manuscript.

Human research participants

Policy information about studies involving human research participants

Population characteristics	Patient-derived tissues were collected for clinical purposes from de-identified brain tumor specimens which is diagnosed with glioblastoma.
Recruitment	Patient-derived tissues were obtained using excess material collected for clinical purposes from de-identified brain tumor specimens. Donors (patients diagnosed with glioblastoma) were anonymous. Progressive numbers were used to label specimens coded in order to preserve the confidentiality of the subjects.
Ethics oversight	This human research with these materials was covered under IRB protocol NCC2019-0241 in National Cancer Center, South Korea.

Note that full information on the approval of the study protocol must also be provided in the manuscript.

Flow Cytometry

Plots

Confirm that:

- [x] The axis labels state the marker and fluorochrome used (e.g. CD4-FITC).
- [x] The axis scales are clearly visible. Include numbers along axes only for bottom left plot of group (a 'group' is an analysis of identical markers).
- [x] All plots are contour plots with outliers or pseudocolor plots.
- [x] A numerical value for number of cells or percentage (with statistics) is provided.

Methodology

Sample preparation	For surface marker analysis, live cells were re-suspended in 0.1% BSA 1xPBS and stained with anti-mouse F4/80 (PE, 1:100, Invitrogen), CD11b (PerCP-Cyanine5.5, 1:100 Invitrogen), and CD86 (APC, 1:100, BioLegend) at 4°C for 20 min. Cells were fixed and permeabilized (Cytofix/cytoperm, BD) for intracellular protein staining, then labeled with anti-mouse CD206 (FITC, 1:100, BioLegend).
Instrument	BD LSRFortessa, BD FACSAria SORP.
Software	FACS Diva and FlowJo software.
Cell population abundance	Purity of post-sort fractions is regularly measured by flow cytometry core facility and >90%.
Gating strategy	For detection of live population were gated by FSC-A/SSC-A. For detection of target population were gated by F4/80(+)CD206 (+) and F4/80(+)CD86(+).

- [x] Tick this box to confirm that a figure exemplifying the gating strategy is provided in the Supplementary Information.
